# Supplementary material for: A neural theory for counting memories
Source: Nat Commun. 2022 Oct 10;13:5961. doi: 10.1038/s41467-022-33577-2 (PMC9551066; doi:10.1038/s41467-022-33577-2)
Supplement: Supplementary file 1 — Supplementary Information [file 41467_2022_33577_MOESM1_ESM.pdf]

A neural theory for counting memories

— Supplementary Information —

Sanjoy Dasgupta[1], Daisuke Hattori[*2], and Saket Navlakha[*3]

[1]University of California San Diego, Computer Science and Engineering Department, La Jolla, CA 92037
[2]University of Texas Southwestern, Department of Physiology, Dallas, TX 75390
[3]Cold Spring Harbor Laboratory, Simons Center for Quantitative Biology, Cold Spring Harbor, NY 11724

[*]Email: daisuke.hattori@utsouthwestern.edu, navlakha@cshl.edu

1. Supplementary Methods

Datasets and pre-processing. The first dataset, Synthetic, consists of $N = 1000$ inputs with $d = 50$ dimensions per input, where each dimension is drawn randomly from an exponential distribution with a fixed mean ($\lambda = 10$). This distribution was selected because several types of neural stimuli, such as faces[1] and odors,[2] are encoded as an exponential distribution of firing rates over a population of neurons, with an approximately fixed mean; i.e., the inputs are encoded using a maximum entropy code.[3] The second dataset, Odors, is experimentally collected response data of $d = 24$ olfactory receptor neurons (ORNs) in the fruit fly to $N = 110$ odors.[4] We fixed the mean of each odor to be constant to mimic the divisive normalization processing that occurs from ORNs to projection neurons in the antennae lobe.[2] This results in an odor representation that is concentration-independent. The third dataset, MNIST, consists of $N = 10000$ images of handwritten digits. Because these raw images consist largely of black pixels, the similarity between many pairs of images, regardless of their class, will be quite high. So, instead of using the raw pixel representation, we trained a LeNet5 network[5] using the 10 class labels. We then extracted a $d = 84$ dimensional feature representation of each image from the inner-most hidden layer in the LeNet5 network. This representation better captured the true similarity structure of digits and resulted in less count interference, compared to the raw input.

Prior to generating the sequence of observations, we reduced each dataset such that there were no pairs of inputs that were highly correlated with each other. Specifically, in each iteration, we selected a random input. If this input did not correlate with any previously kept input, it was kept; otherwise, it was discarded. We iterated until no more inputs could be added. We set the maximum pairwise correlation between any two kept inputs to be no more than 0.80 (for Synthetic, Odors) or 0.70 (MNIST). This process removed no inputs from the Random dataset; it reduced the Odors dataset to $N = 62$ odors; and it reduced the MNIST dataset to $N = 180$ images.

To generate the sequence of observed items, from the reduced dataset of items ($\mathcal{X}$), we drew $n = N$ random samples with replacement according to a Zipf distribution; i.e., in each sampling step, the ith item (ordered arbitrarily) was selected with probability $\propto i^{-a}$, where $a = 0.55$.

For the random matrix M, we used a sparse binary matrix with 6 ones per row, modeling the connectivity between projection neurons and Kenyon cells in the insect mushroom body.[6]

2. Supplementary Note 1: Two counting schemes

(a) The input layer, $x \in \mathbb{R}^d$.

(b) Random projection of the input, $y = Mx \in \mathbb{R}^m$.

Here M is an $m \times d$ random projection matrix whose rows are drawn i.i.d. from some distribution Q. For instance, Q could be the uniform distribution over the d-dimensional unit sphere, or the uniform distribution over all vectors in $\{0,1\}^d$ with exactly c ones, for some small constant c.

We will often look at a single row of the random projection matrix, and denote it by $\theta \in \mathbb{R}^d$.

(c) After winner-take-all operation, $z \in \{0,1\}^m$.

This is given by the rule

$$z_j = \begin{cases} 1 & \text{if } y_j \text{ is one of the } k \text{ largest entries of } y \\ 0 & \text{otherwise} \end{cases}$$

For ease of analysis, we will use an alternative process that produces similar behavior (Section 2.1): it chooses the largest entries of y, and chooses k of them *in expectation.*

(d) Two counting schemes that store information in a vector $w \in \mathbb{R}^m$.

- **Neural count-sketch** (a Hebbian learning rule):
  - Initialize $w_1 = \cdots = w_m = 0$
  - When item x is observed, with tag z: set $w = w + z$

  At any given time, the frequency estimate of an item x, with tag z, is given by $\langle w, z\rangle/k$.
- **1-2-3-many sketch** (an anti-Hebbian learning rule):
  - Initialize $w_1 = \cdots = w_m = 1$
  - When item x is observed, with tag z: set $w_j = w_j e^{-\beta z_j}$ for some constant $\beta > 0$

  At any given time, the 1-2-3-many frequency estimate of an item x, with tag z, is given by $\langle w, z\rangle/k$. This value is roughly 1 if the item has not been seen before (i.e., it is being seen for the first time), $e^{-\beta}$ if it has been seen once before, $e^{-2\beta}$ if it has been seen twice, and less than $e^{-3\beta}$ if it has been seen more often.

2.1 An alternative formulation of winner-takes-all

The winner-take-all operation produces $z \in \{0,1\}^m$ where

$$z_j = \begin{cases} 1 & \text{if } y_j \text{ is one of the } k \text{ largest entries of } y \\ 0 & \text{otherwise} \end{cases}$$

To facilitate the analysis, we will work with an alternative formulation that produces similar results:

$$z_j = \begin{cases} 1 & \text{if } y_j \geq \tau_x \\ 0 & \text{otherwise} \end{cases}$$

Here τ_x is a threshold that is allowed to depend on x. Notice that the rule $(y_j \geq \tau_x)$ indeed picks out the largest entries of y, but might choose more or less than k elements, depending on how τ_x is set. We will specify τ_x so that, in expectation, k entries of y are picked out:

- For a given x and for any fraction $0 < f < 1$, define $\tau_x(f)$ to be the top f-fractile value of the distribution of $\theta \cdot x$, where $\theta \sim Q$, as follows:

$$\tau_x(f) = \sup\{v : \Pr_{\theta \sim Q}(\theta \cdot x \geq v) \geq f\}. \tag{1}$$

  For instance, $\tau_x(1/2)$ is a median value of $\theta \cdot x$.

- Set $\tau_x = \tau_x(k/m)$, so that

$$\Pr_{\theta \sim Q}(\theta \cdot x \geq \tau_x) \approx \frac{k}{m},$$

  where the approximation arises from possible discretization issues. For convenience, we will henceforth assume that this is an exact equality:

$$\Pr_{\theta \sim Q}(\theta \cdot x \geq \tau_x) = \frac{k}{m}. \tag{2}$$

Thus that for any x, the expected number of ones in z is exactly k. Although this τ_x in general depends on x, the scenarios we study have enough symmetry that these thresholds turn out to be the same for all x.

Notice also that this rule is scale-invariant: scaling the x's by a multiplicative constant will simply lead to τ_x being scaled by the same constant.

3. Supplementary Note 2: Theoretical analysis

The method of tag generation effectively induces a similarity function on the input space, $s : \mathcal{X} \times \mathcal{X} \to [0, 1]$. It can be shown that dot products in tag space are, in expectation, proportional to similarity values in $\mathcal{X}$-space. Our analysis makes heavy use of this connection.

3.1 The underlying similarity function

For any two inputs $x, x' \in \mathbb{R}^d$, define

$$s(x, x') = \frac{m}{k} \cdot \Pr_{\theta \sim Q}\left(\theta \cdot x \geq \tau_x \text{ and } \theta \cdot x' \geq \tau_{x'}\right).$$

To interpret this, let $z, z' \in \{0, 1\}^m$ be the projected-and-thresholded versions of x, x', respectively. Then $s(x, x')$ is proportional to the probability that both z_j and z'_j are set to 1, for any specific coordinate $1 \leq j \leq m$; it is scaled by m/k to map it onto the range $[0, 1]$.

The largest possible value of $s(x, x')$ is 1, when $x = x'$. If x' is far from x, in some suitable sense, then $s(x, x')$ will be much smaller. Thus $s(x, x')$ can be thought of as a measure of similarity between x and x'. It is a standard type of similarity function called a *kernel function*, which is another way of saying that it is positive semidefinite.

Lemma 1 *$s : \mathcal{X} \times \mathcal{X} \to \mathbb{R}$ is a kernel function.*

3.2 Similarity function: examples

In earlier work,[7,8] we have derived the form of the similarity function in certain settings.

- *Binary inputs and projection matrix.* Here the inputs are binary vectors with b ones, that is, they lie in the set
$$\mathcal{X} = \{x \in \{0,1\}^d : x \cdot 1 = b\},$$
and the random projection matrix is binary, where Q is the uniform distribution over vectors in $\{0,1\}^d$ that contain exactly c ones, for some integer c. The thresholds τ_x are all set to c; since we need $\Pr_\theta(\theta \cdot x \geq \tau_x) = k/m$, this means that
$$c \approx \frac{\ln(m/k)}{\ln(d/b)}.$$
Then the similarity function has the form
$$s(x, x') \approx \left(\frac{x \cdot x'}{b}\right)^c.$$

- *Gaussian projection matrix.* In this case, the inputs are unit vectors and each entry of the projection matrix is picked from a standard normal distribution; thus Q is $N(0, I_d)$. It can be shown that
$$s(x, x') \approx \left(\frac{k}{m}\right)^{(1 - x \cdot x')/(1 + x \cdot x')}.$$

In both these cases, it can also be seen that when x, x' are chosen independently, $s(x, x') \approx k/m$. It is useful to think of k/m as the similarity value between random independent inputs.

3.3 Dot products in tag-space

In Section 4.2, we show that for any two inputs x, x', their tags Z, Z' (which are random variables because they depend on the random projection matrix M) satisfy
$$\mathbb{E}\langle Z, Z'\rangle = k \cdot s(x, x').$$
Moreover, the actual value of $\langle Z, Z'\rangle$ is tightly concentrated around this expectation. The neural count sketch is based exclusively on dot products in z-space and thus this connection is essential to its analysis. For the 1-2-3-many sketch, we use a different approach.

3.4 The neural count sketch

Suppose that the neural count sketch receives a sequence of n observations in $\mathcal{X}$. What is its frequency response on a subsequent input x?

The notion of a frequency estimate makes the most sense in discrete spaces, whereas in our setting, the input space may well be continuous. One approach to dealing with this is to suitably discretize the space, for instance by focusing on situations in which distinct observations are well-separated from one another. We follow this line of reasoning in Section 3.4.1. In Section 3.4.3, we show that for continuous input spaces the response of the neural count sketch can be seen as a kernel density estimate.

3.4.1 Frequency estimation

Suppose that so far only N distinct observations have appeared, $x^{(1)}, \ldots, x^{(N)}$, and that $x^{(i)}$ has been seen f_i times. Thus the total number of observations, counting duplicates, is $n = f_1 + f_2 + \cdots + f_N$. Given a new input x, we would like the frequency estimate for it to be f_ℓ if $x = x^{(\ell)}$ and 0 if x is different from all the $x^{(i)}$.

A basic difficulty is that the space $\mathcal{X}$ might be continuous and some of the $x^{(i)}$ might be close together. To minimize interference effects, we assume this cannot happen.

Assumption 1 *The n observations seen by the neural count sketch consist of f_i repetitions of $x^{(i)}$, for $i = 1, 2, \ldots, N$. For any $i \neq j$, we have $s(x^{(i)}, x^{(j)}) \leq \xi$, for some $0 < \xi < 1/2$.*

If the distinct $x^{(i)}$ were chosen independently, we'd expect to have $s(x^{(i)}, x^{(j)}) \approx k/m$, as discussed above. Thus the case $\xi = k/m$ is of particular interest.

Theorem 2 *Pick any $0 < \delta < 1$ and define $\sigma = (1/(3k)) \ln(3/\delta)$. Suppose that the neural count sketch witnesses n observations satisfying Assumption 1. Given a subsequent input x:*

(a) The expected response is

$$\sum_{i=1}^{N} f_i s(x, x^{(i)}).$$

(b) If $x = x^{(\ell)}$ then the expected response lies in the interval $[f_\ell, f_\ell + \xi n]$. With probability at least $1 - \delta$, the response is at least $f_\ell(1 - \sqrt{6\sigma})$ and at most

$$f_\ell \left(1 + 3\sqrt{\sigma}\right) + n \left(\xi + \sigma + \sqrt{6\sigma\xi}\right).$$

(c) If x is far from all the $x^{(\ell)}$ in the sense that $s(x, x^{(\ell)}) \leq \xi$ for all $1 \leq \ell \leq N$, then the expected response lies in the interval $[0, \xi n]$. With probability at least $1 - \delta$, the response is at most

$$n \left(\xi + \sigma + \sqrt{6\sigma\xi}\right).$$

To interpret this result, suppose that $\xi = k/m$; as explained above, this is what we would expect if distinct inputs were picked independently. Suppose also that we would like frequency estimates to be accurate within ± 1. For this to hold in expectation, it would be enough to have $m \geq kn$. For it to hold with probability $\geq 1 - \delta$, for frequencies in the range $[0, f]$, it would be sufficient to take $m \geq 2kn$ and $k = \Omega(\max(n, f^2) \ln(1/\delta))$.

In the literature on streaming algorithms, it is common to consider much looser frequency estimates which are accurate within $\pm \epsilon n$ for some constant $\epsilon > 0$. The neural count sketch would achieve this with probability $1 - \delta$ if $m \geq 2k/\epsilon$ and $k = \Omega((1/\epsilon^2) \ln(1/\delta))$.

3.4.2 Bounded precision

Recall that in the count sketch, the weights w_j start off at zero and change only when incremented. We now define a notion of what it might mean for such weights to have "b bits of precision".

Definition 3 *For any positive integers b and $B > 2^b$, we say weights $w_1, \ldots, w_m$ have (b, B)-*precision *if their updates have the following behavior:*

- *If $w_j < 2^b$, then the operation $w_j + 1$ produces the correct value.*
- *If $w_j \geq 2^b$, then the operation $w_j + 1$ results in some value in the range $[w_j, B]$.*

In short, the weights behave linearly in the range $[0, 2^b]$ and then saturate at value B.

Under these conditions, the guarantees of Theorem 2 would continue to hold for queries x with frequency $\leq O(2^b)$, while higher frequencies would get squashed between 2^b and B.

3.4.3 The neural count sketch as a density estimator

The analysis of the previous section applies to a situation where the observations are essentially discrete: any two observations are either duplicates or are far from each other. This is the setting for work on frequency estimation in the streaming algorithms literature.

When the observation space is continuous, it might be more helpful to view the response of the neural count sketch as a *kernel density estimate.* To see how this comes about, suppose that after seeing observations $x^{(1)}, \ldots, x^{(n)} \in \mathcal{X}$, we define

$$\widehat{f}_n(x) = \frac{1}{kn} \langle w, z \rangle,$$

where z is the tag of x. From Lemma 11, we see that this has expectation (over the choice of matrix M)

$$\mathbb{E}_M \widehat{f}_n(x) = \frac{1}{n} \sum_{i=1}^{n} s(x, x^{(i)})$$

and moreover is tightly concentrated around this value. Since each $s(x, x^{(i)})$, seen as a function of x, is continuous, takes values in $[0, 1]$, and is maximized at $x = x^{(i)}$, we can think of it as an unnormalized probability density centered at $x^{(i)}$. Thus $\widehat{f}_n$ is very much like a kernel density estimate, as long as the normalizing constants for the different $s(\cdot, x^{(i)})$ are equal.

Definition 4 *We say that similarity function $s : \mathcal{X} \times \mathcal{X} \to [0, 1]$ has* uniform normalization *if there is a constant $C > 0$ such that for all $x_o \in \mathcal{X}$,*

$$\int_{\mathcal{X}} s(x, x_o)\, \lambda(dx) = C,$$

where λ denotes Lebesgue measure.

The two examples of similarity functions in Section 3.2 both have this property.

3.5 The 1-2-3-many sketch

We now analyze the performance of the 1-2-3-many sketch as a frequency estimator. For an input x, we would hope that the response of the sketch, $\langle W, Z \rangle / k$, is roughly 1 if x has not been seen before, or $e^{-\beta}$ if it has been seen once, or $e^{-2\beta}$ if seen twice, and so on. Equivalently, we can think of the frequency estimate on x as

$$-\frac{1}{\beta} \ln \left(\frac{1}{k} \langle W, Z \rangle \right), \tag{3}$$

rounded to the nearest integer.

3.5.1 Frequency estimation

We study the 1-2-3-many sketch under the same conditions as the neural count sketch (Assumption 1). Recall that a total of n observations are seen, consisting of arbitrarily-interleaved repetitions of N distinct items.

Theorem 5 *Suppose the 1-2-3-many sketch, with parameter* $0 < \beta \leq 1$, *is subjected to* n *observations that satisfy Assumption 1, with* $\xi \leq \beta/(2N)$. *For a subsequent input* $x = x^{(\ell)}$, *the response of the sketch satisfies the following properties.*

(a) The expected response lies in the range $[(1-\beta/2)e^{-\beta f_\ell}, e^{-\beta f_\ell}]$.

(b) Pick any $0 < \delta < 1$. *If* $k \geq (12/\beta^2)\ln(2/\delta)$, *then with probability at least* $1-\delta$, *the response is either* f_ℓ *or* $f_\ell + 1$, *when rounded to the nearest integer.*

This estimate is significantly more accurate than the count-sketch for comparable settings of k and m. First, recall that in order to get good behavior of the count sketch, we needed to take $\xi \leq 1/(2n)$ (or equivalently, $m \geq 2kn$), where n is the total number of observations, including repeats. Here we only need $\xi \leq \beta/(2N)$ (equivalently, $m \geq (2/\beta)kN$), where N is the number of *distinct* observations. Second, for the count sketch, we took $k = O(n)$ to get estimates accurate within ± 1, even for small frequencies. In contrast, here we only need $k = O(1)$.

To understand the shift in dependence from n to N, consider a situation in which there are relatively few distinct observations $x^{(1)}, \ldots, x^{(N)}$, but many repetitions of each. Even if these observations are chosen at random, their tags may still overlap to some extent. Nevertheless, if $\xi \leq c/N$ for some constant $0 < c < 1$, then it is very likely that for any given $x^{(\ell)}$, a constant fraction of the 1's in its tag will not overlap those of the other $N-1$ tags. This pristine part of $x^{(\ell)}$'s tag is what enables the 1-2-3-many sketch to work well, no matter how many times the other observations are repeated. But in the count sketch, the integration of coordinates of Z is done differently and as a result, the noise in the non-pristine part of the tag eventually dominates the frequency estimate.

3.5.2 Bounded precision

Recall that in the 1-2-3-many sketch, all weights w_j start at 1 and are subsequently changed only by multiplication by $e^{-\beta}$. Thus they are monotonically decreasing with time and remain in the range $[0,1]$. We now define a model of bounded precision for such weights.

Definition 6 *For any positive integer* b, *we say weights* $w_1, \ldots, w_m$ *have* b bits of precision *if their updates have the following behavior:*

- *If* $w_j > 2^{-b}$, *then the operation* $w_j e^{-\beta}$ *results in the correct value.*
- *If* $w_j \leq 2^{-b}$, *then the operation* $w_j e^{-\beta}$ *results in a value in the range* $[0, w_j]$.

Under these conditions, the results of Theorem 5 continue to hold for queries with frequency $\leq (b/\beta)\ln 2$. For larger frequencies, the response will be some number greater than or equal to this. Thus if b is at least (roughly) 4β, the 1-2-3-many sketch lives up to its name.

The 1-2-3-many sketch can be seen as a generalization of a Bloom filter, which is in essence a "1-many" sketch: it keeps track of which items are being seen for the first time. The Bloom filter is also based on hashing and has roughly the same complexity, in the sense that it uses $k = O(\log(1/\delta))$ hash functions and a total table size of $m = O(kN)$. It is known that the Bloom filter's resource requirements are optimal within constant factors, and thus the same must hold for the 1-2-3-many sketch.

4. Supplementary Note 3: Proof details

4.1 Proof of Lemma 1

First we observe that the function is symmetric by definition: $s(x, x') = s(x', x)$. Next, pick any finite set of points $x^{(1)}, \ldots, x^{(n)} \in \mathcal{X}$ and $a_1, \ldots, a_n \in \mathbb{R}$. We need to show that

$$\sum_{i,j=1}^{n} a_i a_j s(x^{(i)}, x^{(j)}) \geq 0.$$

To this end, pick $\theta \sim Q$ and define the random variable U_i to be 1 if $\theta \cdot x^{(i)} \geq \tau_{x^{(i)}}$, and 0 otherwise. Then

$$\sum_{i,j=1}^{n} a_i a_j s(x^{(i)}, x^{(j)}) = \frac{m}{k} \sum_{i,j=1}^{n} a_i a_j \mathbb{E}_\theta[U_i U_j] = \frac{m}{k} \sum_{i,j=1}^{n} \mathbb{E}_\theta[a_i a_j U_i U_j] = \frac{m}{k} \cdot \mathbb{E}_\theta \left[\left(\sum_{i=1}^{n} a_i U_i \right)^2 \right] \geq 0.$$

4.2 Dot products in z-space

In what follows, let $z(x, M) \in \{0,1\}^m$ denote the tag for input x if random projection matrix M is used.

Lemma 7 (Expected dot products in z-space) *Fix any $x, x' \in \mathbb{R}^d$. Then,*

$$\mathbb{E}_M[\langle z(x, M), z(x', M) \rangle] = k \cdot s(x, x').$$

PROOF: For fixed x, x', we have

$$\begin{aligned}
\mathbb{E}_M[\langle z(x, M), z(x', M) \rangle] &= \sum_{j=1}^{m} \Pr_M(z(x, M)_j = 1,\ z(x', M)_j = 1) \\
&= \sum_{j=1}^{m} \Pr_{\theta \sim Q}(\theta \cdot x \geq \tau_x,\ \theta \cdot x' \geq \tau_{x'}) \\
&= m \cdot \frac{k}{m} \cdot s(x, x'),
\end{aligned}$$

where we have used the fact that the values of $z(x, M)_j$ and $z(x', M)_j$ depend only on the jth row of M, which is drawn from distribution Q. □

Next, we show that these dot products between tags are tightly concentrated around their expected values. We use a form of Bernstein's inequality[9] from Theorem 2.10 of Boucheron et al.[10] that we reproduce here.

Lemma 8 (Bernstein's large deviation inequality) *Suppose $X_1, \ldots, X_n$ are independent and identically distributed random variables that satisfy $X_i \leq \mathbb{E}X_i + c$ for some constant $c > 0$. Then for any $0 < \delta < 1$, the following holds with probability at least $1 - \delta$:*

$$\sum_{i=1}^{n} X_i - \mathbb{E}\sum_{i=1}^{n} X_i \leq \frac{c}{3}\ln\frac{1}{\delta} + \sqrt{2n \cdot \mathrm{var}(X_i) \cdot \ln\frac{1}{\delta}}.$$

We start with a concentration result that applies to a fixed pair of inputs x, x'.

Lemma 9 (Concentration of dot products in z-space) *Pick any $x, x' \in \mathbb{R}^d$ and any $0 < \delta < 1$. Then with probability at least $1 - \delta$,*

$$\left| \langle z(x, M), z(x', M) \rangle - k \cdot s(x, x') \right| \;\leq\; \frac{1}{3}\ln\frac{2}{\delta} + \sqrt{2k \cdot s(x, x') \cdot \ln\frac{2}{\delta}}.$$

Proof: For $1 \leq j \leq m$, define

$$U_j = \begin{cases} 1 & \text{if } z(x, M) = 1 \text{ and } z(x', M) = 1 \\ 0 & \text{otherwise} \end{cases}$$

Then $\langle z(x, M), z(x', M) \rangle = U_1 + \cdots + U_m$, and by Lemma 7 this has expected value $k \cdot s(x, x')$. The bound follows from observing that the U_j are independent (they depend on different rows of matrix M, chosen independently) and applying the Bernstein bound of Lemma 8. □

Next, we give a concentration result that holds *uniformly* over the entire space $\mathcal{X}$.

Lemma 10 (Uniform concentration of dot-products in z-space) *There is an absolute constant $C > 0$ for which the following holds. Pick any $0 < \delta < 1$. Then with probability at least $1 - \delta$ over the choice of M,*

$$\sup_{x, x' \in \mathcal{X}} \left| \langle z(x, M), z(x', M) \rangle - k \cdot s(x, x') \right| \;\leq\; C(d \ln m + \ln(1/\delta)) + \sqrt{k \cdot s(x, x') \cdot C(d \ln m + \ln(1/\delta))}.$$

Proof: For any $x, x' \in \mathcal{X}$, define $f_{x,x'} : \mathbb{R}^d \to \{0, 1\}$ by

$$f_{x,x'}(\theta) = \begin{cases} 1 & \text{if } \theta \cdot x \geq \tau_x \text{ and } \theta \cdot x' \geq \tau_{x'} \\ 0 & \text{otherwise} \end{cases}$$

This is the intersection of two halfspaces, and thus the class of all these functions, $\mathcal{F} = \{f_{x,x'} : x, x' \in \mathcal{X}\}$, has VC dimension $O(d)$. We can then apply standard relative VC bounds (Theorem 5.1 of Boucheron et al.[11]) to get the following:

- Pick $\theta_1, \ldots, \theta_m \sim Q$ independently
- Let $\widehat{f}_{x,x'} = (f_{x,x'}(\theta_1) + \cdots + f_{x,x'}(\theta_m))/m$

- Then with probability at least $1-\delta$,

$$\sup_{f\in\mathcal{F}} \left| \widehat{f} - \mathbb{E}\widehat{f} \right| \leq \gamma_m + \sqrt{\gamma_m \cdot \mathbb{E}\widehat{f}},$$

  where $\gamma_m = 4(2\mathrm{VC}(\mathcal{F})\ln 2m + \ln(8/\delta))/m$.

In our setting, taking M to be the matrix with rows $\theta_1, \ldots, \theta_m$,

$$\widehat{f}_{x,x'} = \frac{1}{m}\left(f_{x,x'}(\theta_1) + \cdots + f_{x,x'}(\theta_m)\right) = \frac{1}{m}\langle z(x,M), z(x',M)\rangle$$

and thus by Lemma 7 $\mathbb{E}\widehat{f}_{x,x'} = (k/m)s(x,x')$. The lemma statement now follows immediately. □

Finally, we give a specialized concentration bound that applies to the inner product $\langle w, z\rangle$ where w is sum of the tags of n observations.

Lemma 11 (Concentration of frequency response) *Pick any $x^{(1)}, \ldots, x^{(n)}, x \in \mathcal{X}$ and any $0 < \delta < 1$. Suppose matrix M is picked by sampling its rows i.i.d. from distribution Q. Let $Z^{(i)}$ be a shorthand for the random variable $z(x^{(i)}, M)$ and Z for $z(x, M)$. Let $W = Z^{(1)} + \cdots + Z^{(n)}$. Then:*

(a) $\mathbb{E}\langle W, Z\rangle = k\sum_{i=1}^n s(x^{(i)}, x)$.

(b) With probability at least $1-\delta$ over the choice of M,

$$|\langle W, Z\rangle - \mathbb{E}\langle W, Z\rangle| \;\leq\; \frac{n}{3}\ln\frac{2}{\delta} + \sqrt{2n\cdot\mathbb{E}\langle W, Z\rangle \cdot \ln\frac{2}{\delta}}.$$

Proof: Part (a) follows immediately from Lemma 7. For part (b), start by defining $U_j = (Z_j^{(1)} + \cdots + Z_j^{(n)})Z_j$ for $1 \leq j \leq m$. The $U_1, \ldots, U_m$ are independent and identically distributed, with range $0 \leq U_j \leq n$, expected value

$$\mathbb{E}[U_j] = \sum_{i=1}^n \mathbb{E}[Z_j^{(i)} Z_j] = \frac{k}{m}\sum_{i=1}^n s(x^{(i)}, x),$$

and expected squared value

$$\mathbb{E}[U_j^2] = \sum_{1\leq i,i'\leq n} \mathbb{E}[Z_j^{(i)} Z_j^{(i')} Z_j Z_j] \leq \sum_{1\leq i,i'\leq n} \mathbb{E}[Z_j^{(i)} Z_j] = n\cdot\mathbb{E}[U_j].$$

Now, $\langle W, Z\rangle = U_1 + \cdots + U_m$, and the result follows by applying a Bernstein large deviation bound (Lemma 8) to this sum. □

4.3 Proof of Theorem 2

Let $Z^{(i)}$ be a shorthand for $z(x^{(i)}, M)$, the tag assigned to observation $x^{(i)}$ with random projection matrix M. After seeing the n observations, the neural count sketch has weight vector

$$W = f_1 Z^{(1)} + \cdots + f_N Z^{(N)}.$$

Let Z be short for $z(x, M)$, the tag assigned to x.

For part (a), we have from Lemma 11(a) that the expected response of the count sketch on input x is

$$\frac{1}{k}\mathbb{E}\langle W, Z\rangle = \sum_{i=1}^{N} f_i s(x, x^{(i)}).$$

For part (b), we take $x = x^{(\ell)}$ and divide the response into two parts:

$$\frac{1}{k}\langle W, Z\rangle \;=\; \frac{1}{k}\sum_{i=1}^{N} f_i \langle Z^{(i)}, Z\rangle \;=\; \frac{1}{k} f_\ell \|Z^{(\ell)}\|^2 + \frac{1}{k}\sum_{i\neq\ell} f_i \langle Z^{(i)}, Z^{(\ell)}\rangle. \tag{4}$$

Now $\|Z^{(\ell)}\|^2 = Z_1^{(\ell)} + \cdots + Z_m^{(\ell)}$ is the sum of m independent $0-1$ random variables that each have expected value k/m. By a multiplicative Chernoff bound, with probability at least $1 - 2\delta/3$,

$$k\left(1 - \sqrt{\frac{2}{k}\ln\frac{3}{\delta}}\right) \leq \|Z^{(\ell)}\|^2 \leq k\left(1 + \sqrt{\frac{3}{k}\ln\frac{3}{\delta}}\right).$$

The remaining term of (4) is nonnegative and by Lemma 7 has expected value

$$\mathbb{E}\left[\frac{1}{k}\sum_{i\neq\ell} f_i \langle Z^{(\ell)}, Z^{(i)}\rangle\right] \;=\; \sum_{i\neq\ell} f_i s(x^{(\ell)}, x^{(i)}) \;\leq\; n\xi,$$

where the last step invokes Assumption 1. By Lemma 11(b), with probability at least $1 - \delta/3$, this term exceeds its expected value by at most

$$\frac{n}{3k}\ln\frac{3}{\delta} + \sqrt{\frac{2n}{k}\cdot n\xi \cdot \ln\frac{3}{\delta}},$$

whereupon

$$\frac{1}{k}\sum_{i\neq\ell} f_i \langle Z^{(\ell)}, Z^{(i)}\rangle \;\leq\; n\left(\xi + \frac{1}{3k}\ln\frac{3}{\delta} + \sqrt{\frac{2\xi}{k}\ln\frac{3}{\delta}}\right).$$

For part (c), we follow the same reasoning as (b), but focus only on the second term in (4).

4.4 Proof of Theorem 5

In this proof, we'll deal with the more general case where $\xi \leq c/(N-1)$, for some constant $0 < c < 1$, and then specialize later.

Let $Z^{(i)} = z(x^{(i)}, M)$ be a shorthand for the tag of $x^{(i)}$ under random projection matrix M. It follows from the update rule of the 1-2-3-many sketch that after seeing n observations satisfying Assumption 1, the weight vector W has coordinates

$$W_j = \exp\left(-\beta\left(f_1 Z_j^{(1)} + f_2 Z_j^{(2)} + \cdots + f_N Z_j^{(N)}\right)\right).$$

Let $Z = z(x, M)$ be the tag of x. The response on x is then $\langle W, Z\rangle/k$.

Let's analyze this response when $x = x^{(\ell)}$, so that $Z = Z^{(\ell)}$. For any $1 \le j \le m$ with $Z_j = 1$, we have first of all that

$$f_1 Z_j^{(1)} + \cdots + f_N Z_j^{(N)} \ge f_\ell Z_j^{(\ell)} = f_\ell.$$

To upper bound the sum on the left, we look at *how many* of the $Z_j^{(i)}, i \ne \ell$, are set to one.

$$\mathbb{E}\left[\sum_{i\ne\ell} Z_j^{(i)} \middle| Z_j = 1\right] = \sum_{i\ne\ell} \mathbb{E}\left[Z_j^{(i)} \middle| Z_j^{(\ell)} = 1\right] = \sum_{i\ne\ell} s(x^{(i)}, x^{(\ell)}) \le (N-1)\xi \le c.$$

By Markov's inequality, with probability at least $1-c$, this summation is < 1 and hence (since it is integral) zero. If this is true, then we also have $\sum_{i\ne\ell} f_i Z_j^{(i)} = 0$. Hence,

$$\Pr\left(f_1 Z_j^{(1)} + \cdots + f_N Z_j^{(N)} = f_\ell \middle| Z_j = 1\right) \ge 1 - c.$$

Thus whenever $Z_j = 1$, we have $W_j \le e^{-\beta f_\ell}$ and $\Pr(W_j = e^{-\beta f_\ell} | Z_j = 1) \ge 1 - c$.

We can now show part (a) of theorem statement.

$$\mathbb{E}\left[\frac{1}{k}\langle W, Z\rangle\right] \;=\; \frac{1}{k}\sum_{j=1}^{m} \Pr(Z_j = 1)\mathbb{E}[W_j | Z_j = 1] \;=\; \frac{1}{m}\sum_{j=1}^{m} \mathbb{E}[W_j | Z_j = 1].$$

We have seen that $\mathbb{E}[W_j|Z_j = 1]$ lies in the range $[(1-c)e^{-\beta f_\ell}, e^{-\beta f_\ell}]$. The stated bound follows by taking $c = \beta/2$.

Now we move on to the large-deviation bound for part (b). Let V_j denote the event that both $Z_j = 1$ and $W_j = e^{-\beta f_\ell}$. Then:

- $Z_1, \ldots, Z_m$ are i.i.d. with $\mathbb{E}[Z_j] = k/m$
- $V_1, \ldots, V_m$ are i.i.d. with $\mathbb{E}[V_j] \ge (1-c)k/m$

Take any $0 < \delta < 1$ and $0 < \epsilon \le 1/2$. We can apply Chernoff bounds to show that with probability at least $1 - \delta$,

$$Z_1 + \cdots + Z_m \le (1+\epsilon)k \qquad (E_1)$$

$$V_1 + \cdots + V_m \ge (1-\epsilon)(1-c)k \qquad (E_2)$$

provided $k \ge (3/\epsilon^2)\ln(2/\delta)$. Under event (E_1), the response can be upper-bounded by

$$\frac{1}{k}\langle W, Z\rangle \le \frac{1}{k}\sum_{j=1}^{m} Z_j e^{-\beta f_\ell} \le (1+\epsilon)e^{-\beta f_\ell},$$

while under event (E_2), we get a lower bound on the response,

$$\frac{1}{k}\langle W, Z\rangle \geq \frac{1}{k}\sum_{j=1}^{m} V_j e^{-\beta f_\ell} \geq (1-\epsilon)(1-c)e^{-\beta f_\ell}.$$

The corresponding frequency estimate (3) lies in the interval

$$\left(f_\ell - \frac{\epsilon}{\beta},\ f_\ell + \frac{3}{2\beta}(c+\epsilon)\right).$$

If ϵ and c are both $\leq \beta/2$, then this value is either f_ℓ or $f_\ell + 1$ when rounded to the nearest integer.

References

[1] Stevens, C. F. Conserved features of the primate face code. *Proc Natl Acad Sci U S A* **115**, 584–588 (2018).

[2] Stevens, C. F. What the fly's nose tells the fly's brain. *Proc Natl Acad Sci U S A* **112**, 9460–9465 (2015).

[3] Stevens, C. F. A statistical property of fly odor responses is conserved across odors. *Proc Natl Acad Sci U S A* **113**, 6737–6742 (2016).

[4] Hallem, E. A. & Carlson, J. R. Coding of odors by a receptor repertoire. *Cell* **125**, 143–160 (2006).

[5] Lecun, Y., Bottou, L., Bengio, Y. & Haffner, P. Gradient-based learning applied to document recognition. *Proceedings of the IEEE* **86**, 2278–2324 (1998).

[6] Caron, S. J., Ruta, V., Abbott, L. & Axel, R. Random convergence of olfactory inputs in the drosophila mushroom body. *Nature* **497**, 113–117 (2013).

[7] Dasgupta, S., Sheehan, T. C., Stevens, C. F. & Navlakha, S. A neural data structure for novelty detection. *Proc Natl Acad Sci U S A* **115**, 13093–13098 (2018).

[8] Shen, Y., Dasgupta, S. & Navlakha, S. Habituation as a neural algorithm for online odor discrimination. *Proc Natl Acad Sci U S A* **117**, 12402–12410 (2020).

[9] Bernstein, S. *The Theory of Probabilities* (Gastehizdat Publishing House, Moscow, 1946).

[10] Boucheron, S., Lugosi, G. & Massart, P. *Concentration Inequalities: A Nonasymptotic Theory of Independence* (Springer, 2013).

[11] Boucheron, S., Bousquet, O. & Lugosi, G. Theory of classification: a survey of recent advances. *ESAIM: Probability and Statistics* **9**, 323–375 (2005).
